# COVID-19 infections, recoveries, and mortality: an ANOVA model of locations and administrative areas in Saudi Arabia

**DOI:** 10.3389/fpubh.2024.1281289

**Published:** 2024-01-17

**Authors:** Hamad Mansur Aldossari, Asharaf Abdul Salam

**Affiliations:** ^1^Geography and Geographical Information Systems Department, College of Social Sciences, Imam Mohammad Ibn Saud Islamic University (IMSIU), Riyadh, Saudi Arabia; ^2^King Saud University, Center of Population Studies, Riyadh, Saudi Arabia

**Keywords:** univariate general linear model, urbanization, infrastructure development, mean number of infected cases, grassroot level interventions

## Abstract

**Background:**

Saudi Arabia has 13 administrative areas, all of which have been seriously affected by the COVID-19 epidemic regardless of their features. Being the largest and a prominent Arab country, epidemic intensity and dynamics have importance, especially in the era of Vision 2030 where infrastructure development and growth to enhance quality of life has of prime focus.

**Aims:**

This analysis aims to trace the differentials in COVID-19 infections, recoveries, and deaths across the country depending upon various demographic and developmental dimensions and interactions.

**Data and methods:**

This analysis used Saudi Arabia Ministry of Health data from March 15th, 2020 to August 31st, 2022, by classifying administrative areas and locations to build a generalized linear model (3 × 3): three types of administrative areas (major, middle-sized, and others) and localities (major, medium-sized, and others). Apart from two-way ANOVA, an one-way ANOVA also carried out in addition to calculating mean values of infections, recoveries, and deaths.

**Results:**

A total of 205 localities were affected with varying severity, which are based on local demographics. Both the administrative areas and localities had a significant number of cases of infections, recoveries, and mortality, which are influenced by relationships and interactions, leading to differential mean values and proportional distributions across various types of administrative areas and localities.

**Conclusion:**

There is dynamism that major administrative areas have lesser threats from the epidemics whereas medium-sized ones have serious threats. Moreover, an interaction of administrative areas and localities explains the dynamics of epidemic spread under varying levels of infrastructure preparedness. Thus, this study presents lessons learned to inform policies, programs, and development plans, especially for regional, urban, and infrastructure areas, considering grassroots level issues and diversity.

## 1 Introduction

With almost 35 million people living in an area of approximately 2 million square kilometers, the rapid spread of COVID-19 in Saudi Arabia underwent an initial rapid rise and peak, followed by further erratic behavior during the three years period of the epidemic. Geographic variations occurred that were dependent on super-spreaders and population heterogeneity, local and regional based on various socioeconomic and demographic conditions (1–4). Highly populated industrial cities demanded epidemic compartmentalization models for policy interventions, distinguishing early implementation, later interventions, and mild interventions to pave the way for post-pandemic urban growth strategies and potential (5, 6). Such compartmentalized units are common in Saudi Arabia, where the spatial architecture, urbanism, and local governance patterns differ, adding to the challenges of designing and implementing protective measures in times of emergencies including epidemics (7). In addition to regional comparisons of COVID-19 infection, mortality determined by health service delivery, community-level healthcare, testing approaches, and the characteristics of surveillance systems are dynamics that provide opportunities to balance regional development (6, 8).

Despite the precautionary and preventive measures adopted in Saudi Arabia, the epidemic spread faster than expected, necessitating proactive precautionary political and economic strategies to flatten the epidemic curve by increasing recovery to achieve a comparatively low case fatality rate, the burden of disease (9–12). In addition, the country continued decisive bold steps to safeguard the population at an immense socioeconomic cost, implementing swift community action and hospital preparedness, to increase the impact of mitigation measures (4, 10, 13–16). As a result, seriously affected metropolises and future cities having an availability of essential health services emerged with models of care and protective measures to contain the epidemic (11). On the other hand, poorly developed urban centers (towns and cities) had manifold challenges. Rapid urbanization, particularly around cities with migrant load, demands a rethinking beyond conventional neoliberal strategies to reduce risks of epidemics and other emergencies considering standards of population, space, and utilities to ensure quality of living arrangements through national, regional, and local planning, design, and development strategies (6, 17).

Cities with high population concentrations and economic pressures were seriously affected and were considered as COVID-19 hotspots. The dynamics of the pandemic in urban areas posed critical threats of COVID-19 but with variations of available combating infrastructure as determinants of preparedness (18). The large geographic area of Saudi Arabia has varied climatic and ecological conditions, climatic sensitivity, socioeconomic conditions, seasonal patterns, and population factors that played havoc in affecting transmission, creating disease burden and comorbidity, and warranting control policies (11, 19, 20). Such a vast geographic area leads to limitations in access to healthcare professionals, delays between disease development, progression, and diagnosis, and poor information concerning disease spread due to the absence of trustworthy sources and reliable guidance in emergencies (21, 22). Saudi Arabian locations vary in terms of population dynamics, geography, environments, and resource availability; health infrastructure such as hospitals and medical facilities producing varying levels of accessibility, and risk factors, immune system responses, responses to treatment, and danger of mortality make areas either safe, susceptible, or vulnerable (23). Thus, there is a heterogeneity of population and geographic characteristics making uniform effects as less impactful.

COVID-19 infections are invasions and multiplications of microorganisms, and viruses, that are normally present in the body. This sickness is caused by a virus called Sever Acute Respiratory Syndrome Coronavirus 2. Recoveries from COVID-19, on the other hand, refers to a probable confirmed case known to be alive and 14 days have elapsed. Death from COVID-19 is defined as a death resulting from a clinically compatible confirmed case, unless there is a clear alternative cause of death, not related to COVID-19 disease.

Thus, it makes imperative to have analyses and interpretations separating localities on various dimensions of geography, population, and infrastructure. This study aimed to test a model comparing three administrative area types and three types of neighborhoods in terms of differentiation of COVID-19 infections, recoveries, and deaths reported daily, examining the various interactions and relationships involved. It is hypothesized that major administrative areas having well laid out infrastructure have all strengths to safeguard population from threatening situations, although with a buzzer period. While the upcoming fast changing geographic/administrative areas have challenges of meeting demands posed by epidemic emergencies, the other geographic/administrative areas and localities have lesser impact and demand.

### 1.1 Data

This study used daily reports of COVID-19 infection published by the Saudi Arabian Ministry of Health, from March 15, 2020 to August 31, 2022. Data were compiled in Excel sheets and analyzed using SPSS 25. The data analyzed refers to the entire cases of infections, recoveries, and deaths. There are no sampling methods involving inclusions or exclusions adopted. At least one person was infected in 205 locations during the studied period; 100 were in major administrative areas (Riyadh, Makkah Al-Mokarramah, Al-Madina Al-Monawarah, and the Eastern Region), 55 were in middle-sized areas (Qassim, Aseer, Northern Borders, and Najran), and 50 were in other administrative areas (Tabuk, Hail, Jazan, Al-Baha, and Al-Jouf). Of all locations, 13 were administrative capitals, 61 were medium-sized locations (grade A governorate headquarters), and 131 were other types, usually smaller and remote locations.

### 1.2 Statistical analyses

The above mentioned combination of administrative areas and localities formed a 3 × 3 model comprising of 3 types of administrative areas (large, middle-sized, and others) and 3 types of localities (large medium-sized, and others). Such an analysis applies to both normally distributed and non-normally distributed data as they provide valid outcomes explaining the effect of two categorical variables on the dependent variable. This major analysis of the univariate general linear model (Two-Way ANOVA) was carried out to find out the effect of administrative areas and localities separately and thereby the interaction effect of both these variables. To be specific, the interaction effect represents the combined effects of factors on dependent variable, that is, dependence of the impact of one factor on the level of the other.

In addition to this main analysis, locations with at least one person infected, recovered, or dead in each category were extracted. The number of infected, recovered, and dead persons in every administrative area was also analyzed. One-way ANOVA of cases (infections, recoveries, and deaths for years 2020, 2021, and 2022, and the total) was performed with the administrative areas and localities, each classified into three types. The mean number of infections, recoveries, and deaths in each category for the locality and administrative area was assessed. Analyses by years were also performed for all the aforementioned indicators. Significance levels of *p* < 0.05 (Five percent) was set as acceptable. Graphs are prepared by keeping the proportion of cases (infections, recoveries, and deaths) by type of locations (total of the three types of locations add to 1).

## 2 Results

**Figure 1:**
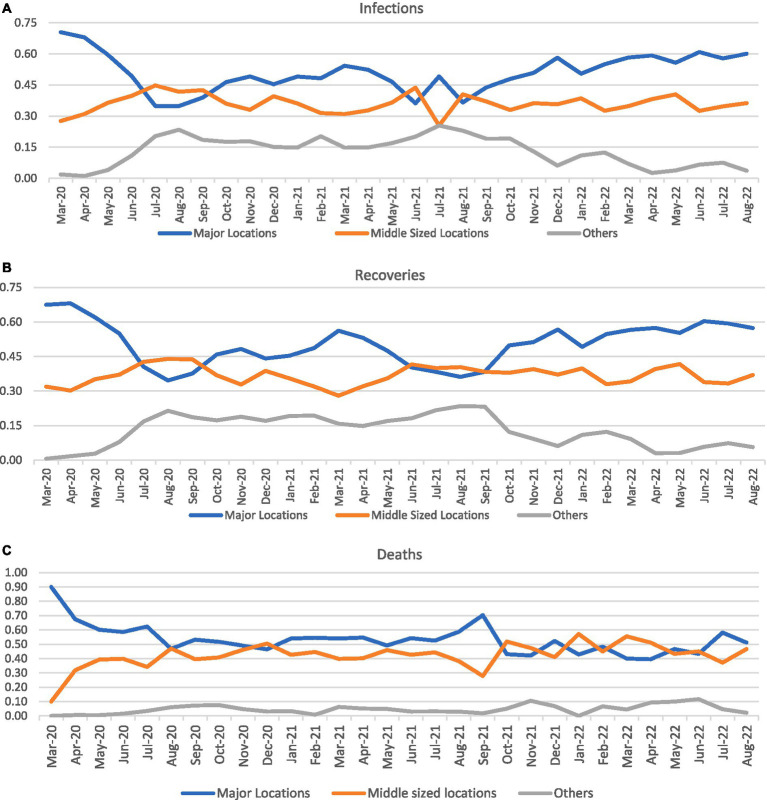
Proportion of cases by month in various categories of locations.

Overall, 205 locations had at least one infected person, 204 locations had at least one person who recovered, and 99 locations had at least one person who died (Tables 1A,B). In the first category, 99 were from major administrative areas, 56 were from middle-sized administrative areas, and 50 were from smaller areas. The corresponding figures for the second and third variables were 99, 55, and 50 and 42, 27, and 30, respectively. The annual distributions of these numbers are shown in Tables 1A,B, along with the total number of infections, recoveries, and deaths in each type of administrative area. Since these infected locations have a rapidly developing infrastructure, their COVID-19 cases multiplied geometrically, as revealed by increased infections, recoveries, and deaths. The other 131 smaller locations also experienced rapidly increasing infections, of which 130 locations recorded recoveries and 38 reported deaths. Even these smaller localities have comparatively lower number reporting a death (Figure 1). In short, while the larger locations in major regions had a monthly decline in reported cases, there was a faster and slower increase in the middle-sized and smaller locations, respectively. This trend is probably reflected in the interaction results as well.

**Table 1A:** Number of neighborhoods having at least one person infected, recovered or died.

Neighborhoods	Infected				Recovered				Died			
	2020	2021	2022	Total	2020	2021	2022	Total	2020	2021	2022	Total
**Major administrative areas**												
Large Medium-sized Others Total	4 31 63 98	4 31 57 92	4 31 57 92	4 31 64 99	4 31 64 99	4 31 58 93	4 31 57 92	4 31 64 99	4 19 15 38	4 18 7 29	4 11 1 16	4 22 16 42
**Middle-sized administrative areas**												
Large Medium-sized Others Total	4 14 37 55	4 14 36 54	4 14 36 54	4 14 38 56	4 14 37 55	4 14 34 52	4 14 35 53	4 14 37 55	4 10 8 22	4 10 8 22	4 4 6 14	4 13 10 27
**Other administrative areas**												
Large Medium-sized Others Total	5 16 29 50	5 16 29 50	5 16 29 50	5 16 29 50	5 16 29 50	5 16 29 50	5 16 29 50	5 16 29 50	5 11 11 27	5 10 7 22	5 8 2 15	5 13 12 30
**Total**												
Large Medium-sized Others Total	13 61 129 203	13 61 122 196	13 61 122 196	13 61 131 205	13 61 130 204	13 61 121 195	13 61 121 195	13 61 130 204	13 40 34 87	13 38 22 73	13 23 9 45	13 48 38 99

**Table 1B:** Number persons included in the analysis by administrative areas classified.

Major				Middle Sized				Others			
Name (Number of Locations)	Infected	Recovered	Died	Name (Number of Locations)	Infected	Recovered	Died	Name (Number of Locations)	Infected	Recovered	Died
**2020**											
Riyadh (33) Makkah Al Mokarramah (29) Al Madina Al Monawarah (11) Eastern Region (27) Total (100)-	75,126 88,030 29,835 88,195 281,186 -	73,380 85,319 29,439 86,964 275,102 -	1,251 2,375 163 849 4,638 -	Qaseem (14) Aseer (25) Northern Borders (8) Najran (7) Total (54) -	13,914 28,281 2,491 6,513 51,199 -	13,592 27,690 2,223 6,376 49,881 -	195 441 87 66 789 -	Tabuk (8) Hail (9) Jazan (17) Al Baha (9) Al Jouf (8) Total (51)	5,043 7,317 12,099 4,511 1,290 30,260	4,895 7,090 11,503 4,330 1,176 28,994	85 131 466 666 55 803
**2021**											
Riyadh (33) Makkah Al Mokarramah (29) Al Madina Al Monawarah (11) Eastern Region (27) Total (100) -	59,065 45,146 10,272 30,898 145,381 -	57,646 42,316 9,962 30,137 140,061 -	322 827 155 501 1805 -	Qaseem (14) Aseer (25) Northern Borders (8) Najran (7) Total (54) -	7,593 13,114 2,841 4,222 27,770 -	7,427 12,723 2,841 4,183 27,174 -	99 235 26 50 410 -	Tabuk (8) Hail (9) Jazan (17) Al Baha (9) Al Jouf (8) Total (51)	3,410 4,435 9,089 2,719 1,419 21,072	3,319 4,386 8,876 2,735 1,372 20,688	28 93 214 32 66 433
**2022**											
Riyadh (33) Makkah Al Mokarramah (29) Al Madina Al Monawarah (11) Eastern Region (27) Total (100) -	86,753 66,036 15,349 41,312 209,450 -	86,927 67,553 15,266 41,678 211,424 -	30 90 10 72 202 -	Qaseem (14) Aseer (25) Northern Borders (8) Najran (7) Total (54) -	7,584 12,891 1,604 2,883 24,962 -	7,649 12,863 1,640 2,884 25,036 -	15 103 8 10 136 -	Tabuk (8) Hail (9) Jazan (17) Al Baha (9) Al Jouf (8) Total (51)	3,698 2,852 11,003 3,359 1,188 22,100	3,712 2,859 10,993 3,368 1,211 22,143	9 14 38 12 8 81
Total											
Riyadh (33) Makkah Al Mokarramah (29) Al Madina Al Monawarah (11) Eastern Region (27) Total (100) -	220,944 199,212 55,456 160,405 636,017 -	217,953 195,188 54,667 158,779 626,587 -	1,603 3,292 328 1,422 6,645 -	Qaseem (14) Aseer (25) Northern Borders (8) Najran (7) Total (54) -	29,091 54,286 6,936 13,618 103,931 -	28,668 53,276 6,704 13,443 102,091 -	309 779 121 126 1,235 -	Tabuk (8) Hail (9) Jazan (17) Al Baha (9) Al Jouf (8) Total (51)	12,151 14,604 32,191 10,589 3,897 73,432	11,926 14,335 31,372 10,433 3,759 71,825	122 238 718 110 129 1,317

Considering the research theme of this, the data were analyzed using a 3 × 3 ANOVA (univariate general linear model) for localities as classified. Significant *F*-values were found for locations, administrative areas, and their interactions (*p* < 0.001) for infections, recoveries, and deaths (Table 2). Of the considered localities, divided into large administrative capitals, medium-sized headquarters of A-type governorates, and others (small towns and villages) located in 13 administrative areas (divided into major, medium-sized, and others). Both the broader administrative boundaries and the location characteristics impacted the cases during the COVID-19 epidemic.

**Table 2:** Two-Way ANOVA results (3×3 Model) calculated with univariate general linear model.

Month	F (Significance)					*R*^2^ (Adjusted r^2^)
	Corrected Model	Intercept	Admin; area	Neighbor-hoods	Admin; area X location	
**Infections**						
2020	41.353*	175.770*	75.143*	87.836*	42.286*	0.630 (0.615)
2021	15.370*	70.860*	28.747*	32.158*	15.372*	0.397 (0.371)
2022	17.530*	64.115*	29.812*	38.446*	19.226*	0.429 (0.404)
Total	25.838*	105.810*	46.244*	55.273*	27.042*	0.513 (0.493)
**Recoveries**						
2020	42.565*	179.538*	76.877*	90.821*	43.768*	0.635 (0.620)
2021	15.661*	72.690*	29.557*	32.463*	15.618*	0.390 (0.365)
2022	18.631*	67.880*	31.725*	40.859*	20.488*	0.432 (0.409)
Total	25.856*	105.660*	46.143*	55.445*	27.114*	0.513 (0.494)
**Deaths**						
2020	16.390*	78.748*	37.166*	27.934*	13.414*	0.401 (0.376)
2021	31.861*	175.526*	81.012*	45.303*	22.315*	0.565 (0.548)
2022	18.918*	152.284*	60.924*	11.411*	7.284*	0.436 (0.413)
Total	20.828*	106.502*	49.569*	33.049*	16.062*	0.460 (0.437)

*p < 0.001.

The *F* value of location was found to be the highest for both infections (55.273), recovery (55.445), and deaths (33.049). Higher *F* values were reported during the intense spread of COVID-19, though the *F* value for the administrative area remained lower throughout the study period, except for that of deaths. This pattern was also reflected in the interactions, indicating that the two variables worked together to lead to different COVID-19 infection statistics. Of the reasonable R^2^ values found, most were above 0.40 and the adjusted R^2^ was above 0.30, indicating that the tests were logical showing the proportion of variance explained. These results shows agreement with the hypothesis that the COVID-19 varied across administrative areas and localities differentiated on the basis of infrastructure and pace of development. It also shows how both these indicators operate together to create a difference. There are localities of various types within an administrative area and so the potential of an administrative area cannot alone explain the intensity of COVID-19 spread.

All the 13 urban pockets and administrative capitals of Riyadh, Makkah Al-Mokarramah, Al-Madina Al-Monawarah, Dammam, Buraydah, Abha, Arar, Najran, Tabuk, Hail, Jazan, Al-Baha, and Sakaka accounted for a large share of the cases initially, which declined rapidly over the studied period. While the major locations, the administrative area headquarters (13 in number) stands as the highest infected, recovered and dead localities, the middle-sized localities (A grade governorate headquarters) have had cases reaching to the former category accounting at certain points in case of locations than administrative areas (46.244 against 55.273 in case of infections and 46.143 against 55.445 in case of recoveries). The reverse is true in the case of deaths (49.569 against 33.049). Higher the *F* ratio greater the significance, although the values are highly significant.

An One-Way ANOVA was executed to examine infected cases, recoveries, and deaths, considering administrative areas and locations as independent variables, and assessing their individual impacts. The former was not found to be significant at *p* = 0.05, except for infections in 2020, while the latter variable was significant (*p* < 0.001) throughout the period for all three indicators (Table 3). These results indicated that more than the broader administrative areas, smaller homogeneous geographic units and locations played prominent roles in increasing the spread of COVID-19 and thereby recoveries and mortality. During the initial stages of infection, administrative areas did not play significant roles, but their roles slowly became clearer; by 2022. With time, these areas started to play prominent roles in the epidemic spread, which explains the geographic variations in the spread of COVID-19, along with population heterogeneity.

**Table 3:** Results One-Way ANOVA (*F* Values) of COVID-19 year wise with administrative area (3 groups) and locations (3 groups).

years	Administrative area	Locations
**Infections**		
2020	3.119**	34.983*
2021	2.083	19.272*
2022	2.054	18.698*
Total	2.399	26.367*
**Recoveries**		
2020	3.152**	35.373*
2021	1.842	20.133*
2022	1.908	19.900*
Total	2.414	26.268*
**Deaths**		
2020	1.388	27.218*
2021	1.316	52.189*
2022	0.224	52.658*
Total	1.345	34.949*

*p < 0.001, **p < 0.05.

An analysis of the mean number of infected cases was performed using the classification adopted in the 3 × 3 design (Table 4) for a better clarity, thus allotting a mean for each category of location (administrative area capital, A Grade governorate headquarters, and others) in each type of administrative area (major, medium-sized, and others). Each location, as a whole, had 3,967 infected persons, 3,905 recoveries, and 45 deaths, at the end of August 2022. A large share of these infections, recoveries, and mortality were recorded at the end of 2020 (the first wave) although the subsequent waves had not created such a heavy burden.

**Table 4:** The mean number of infected persons in each category of neighborhood by administrative area classified, month-wise.

	Infections				Recoveries				Deaths			
Regions/locations	2020	2021	2022	Total	2020	2021	2022	Total	2020	2021	2022	Total
**Major regions**												
Major locations Medium sized locations Others Total	34,444 3,642 2,868	17,103 1,812 365 1,580	29,297 2,471 277 2,278	80,844 7,925 1,047 6,424	33,735 3,559 465 2,778	16,303 1,744 325 1,415	29,538 2,500 249 2,137	79,576 7,802 1,039 6,330	608 66 3 47	236 26 1 18	25 3 0 2	869 95 3 67
**Middle-sized regions**												
Major locations Medium-sized locations Others Total	4,203 1,490 373 936	2,425 674 246 518	2,569 625 169 465	9,198 2,788 756 1,867	4,036 1,458 357 895	2,366 660 229 489	2,589 626 160 450	8,991 2,744 746 1,834	119 19 1 14	58 11 1 7	11 5 1 2	189 34 3 24
**Others**												
Major locations Medium-sized locations Others Total	3,011 655 155 601	1,634 440 194 417	2,020 506 125 436	6,664 1,601 474 1,454	2,876 632 150 576	1,586 431 194 409	2,024 505 125 436	6,487 1,567 468 1,422	102 15 2 16	51 9 1 9	9 2 0 2	163 26 3 26
**Total**												
Major locations Medium-sized locations Others Total	13,049 2,365 377 1,786	6,637 1,191 289 991	10,582 1,532 209 1,309	30,269 5,087 836 3,967	12,728 2,309 364 1,727	6,354 1,151 268 917	10,664 1,546 196 1,261	29,746 5,006 828 3,905	263 42 2 30	110 18 1 13	15 3 0 2	388 63 3 45

In major administrative areas, the mean numbers of infected, recovered, and dead persons were 6,424, 6,330, and 67, respectively at the end of the period of analysis; the corresponding figures for medium-sized areas were 1,867, 1,834, and 24, and for others were 1,454, 1,422, and 26, respectively. These figures vary through the years and also through types of administrative areas and localities.

## 3 Discussion

An imminent need to rethink the conventional growth strategies of cities in line with growth models and urbanization regards emergency preparedness and epidemic spread in planning, design, and development strategies (3, 6). COVID-19 has informed the community, development planners, and policymakers of the integral transformative actions for creating resilient and sustainable cities (18). It is also imperative for authorities to be vigilant to provide evidence in preparation for immediate intervention measures and policies in case of epidemics and emergencies (19).

Although the major administrative areas accounted for the majority of infected cases, rapid actions, interventions, and infrastructure turned these higher numbers into recoveries. But the more homogenous neighborhoods characterized by demographics, geography, livelihoods, and expatriate population proportion had serious repercussions of super-spreaders. Thus, conflicting scenarios have resulted due to the preparedness in the rapidly developing centers and geographic clusters (1).

The variables, assessed in a 3 × 3 model, significantly affected the number of COVID-19 cases, similar to the epidemic compartmental model (5). The high population concentration and economic activities in urban areas made them hotspots for COVID-19, as revealed by its worldwide spread at various locations (18). These locations differ in climatic conditions, socioeconomic status, and disease control and containing capacity, determining the transmission rates and thus the health status (19).

The higher levels of mitigation potential of metropolitan cities with high community and business activities have higher emergency preparedness, especially in health delivery, community outreach, and diagnostic and surveillance systems (8). During the early months of infection (March and April 2020), these major cities had higher proportions (more than 80%) of all cases, which decreased to 48% by November of the same year. Whether adopting intervention strategies earlier or later impacts effectiveness should still be examined, in addition to characteristics such as spatial architecture and governance patterns (5, 7). Saudi Arabia has taken the lead in implementing precautionary measures to anticipate the dangers of the epidemic throughout the country (9); however, emergencies and diseases spread to all parts of the country with varying intensities (6). Because of this, explaining the opportunities and dynamics at the local level is essential, especially along future city programs initiated in the country that are moving ahead progressively.

The impact of environmental quality, socioeconomic impacts, management and governance, and transportation and urban design on the epidemic spread is being discussed (18, 24). These impacts are also affected by super-spreaders, as previously stated (1, 2, 5). This calls for human settlements to seek options to distribute populations, including migrants, to various parts of the country (6). The digital health declaration enacted to address health needs virtually smoothed the implementation of preventive and curative programs (16). Mitigation strategies at venues where people meet a large number of strangers should be mandatory (1). In addition, different strategies and restrictions adopted should be tailored to population density, considering the employment and vulnerability of marginal populations (3, 17, 25, 26).

## 4 Conclusions and recommendations

The popular concept of giving importance to grassroots-level action plans and interventions to give output was accurate in the context of the COVID-19 epidemic in Saudi Arabia. The larger administrative areas having density of population, administration and policy implementation had high potential and preparedness to address emergencies, especially healthcare interventions to address COVID-19. The number of infected cases changed in a haphazardous manner due to uncontrolled factors; however, the increasing number of infections started to follow a pattern inside the middle-sized administrative areas, which emerged as hostile factors. In contrast, smaller neighborhoods had less impact of infected cases and thus recoveries and mortality. The interaction between neighborhoods and administrative area dynamics was significant, indicating an importance of these interactions in causing infections in combination with administrative area and locality types. Thus, developmental plans, programs, and policies taking grassroots-level demographic dynamics into account are valuable, especially in the context of epidemics and emergencies. And so, more and more analyses are needed to extract grassroot level factors by exploring national databases.

## Author contributions

HA: Conceptualization, Data curation, Writing – original draft, Writing – review & editing.

AS: Formal analysis, Supervision, Writing – review & editing.
